# Association between Dynamic Contrast-Enhanced MRI Parameters and Prognostic Factors in Patients with Primary Rectal Cancer

**DOI:** 10.3390/curroncol30020194

**Published:** 2023-02-20

**Authors:** Hye Ri Kim, Seung Ho Kim, Kyung Han Nam

**Affiliations:** 1 Department of Radiology, Inje University College of Medicine, Haeundae Paik Hospital, Busan 48108, Republic of Korea; 2 Department of Pathology, Inje University College of Medicine, Haeundae Paik Hospital, Busan 48108, Republic of Korea

**Keywords:** rectum, cancer, dynamic contrast enhanced magnetic resonance imaging, tumor differentiation, prognosis

## Abstract

Background: To evaluate the association between perfusion parameters derived from dynamic contrast-enhanced magnetic resonance imaging (DCE-MRI) with prognostic factors in primary rectal cancer patients. Methods: A sample of 51 patients with pathologically proven rectal adenocarcinoma through surgery were retrospectively enrolled. All the patients underwent preoperative DCE-MRI including 3D-spoiled gradient echo. Two radiologists determined the tumor border after radiologic–pathologic correlation and drew regions of interest. The perfusion parameters, including the volume transfer constant (K^trans^), were calculated under the extended Toft model. The prognostic factors included TN stage, circumferential resection margin, extramural venous invasion, Kirsten-ras mutation, tumor size, carcinoembryonic antigen, and tumor differentiation. The association was assessed via correlation or t-test. For significant prognostic factors, receiver operating characteristic (ROC) curve analyses were performed to estimate the diagnostic predictive values. Results: K^trans^ only showed a significant difference according to tumor differentiation, between the well-differentiated (*n* = 6) and moderately differentiated (*n* = 45) groups (0.127 ± 0.032, 0.084 ± 0.036, *p* = 0.036). The AUC was 0.838 (95% CI, 0.702–0.929), and the estimated accuracy, sensitivity, and specificity were 87%, 90%, and 60%, respectively. Conclusions: K^trans^ showed a significant difference based on tumor differentiation, which may be conducive to prediction of prognosis in primary rectal cancer.

## 1. Introduction

Rectal cancer is ranked third among the most common causes of death associated with malignancy and is one of the cancers that require a personalized treatment approach [1]. Almost 70% of patients are over 65 years of age and the 5-year survival rates after surgical excision are known to be 85–95% for stage I and less than 10% for stage IV cancer [2]. At the time of diagnosis, liver metastases account for 15–25% of colorectal cancer patients, which is one of the causes of high mortality [3]. It is known that some colorectal cancer patients with liver metastases can be cured with liver resection; however, the rate has been reported to be less than 20–30%. Therefore, combined chemotherapy including targeted therapy, with surgery has been attempted to cure the disease [4]. Like other cancers, rectal cancer is caused by genetic alterations that alter normal cell function. Spontaneous mutations—such as KRAS, APC, and TP53—are known to contribute to the development and progression of diseases. Using this, targeted therapies that specifically target mutations, which have fewer side effects and are more effective than generalized cancer treatments, are used for cancer treatment [5]. These genetic factors as well as clinico-pathological factors such as tumor stage, node stage, extramural venous invasion (EMVI), serum carcinoembryonic antigen (CEA) levels, tumor differentiation, and tumor size are known to be useful indicators for predicting prognosis [6,7]. Therefore, studies have been conducted to attempt to find associations between radiologic findings and these alleged prognostic factors [2,8,9,10].

Conventional MRI could be useful in identifying tumor location, morphology, size, tumor and lymph node stages, and detecting the presence of EMVI. Patients with locally advanced rectal cancer on MRI (≥T3 or node-positive) benefit from neoadjuvant chemoradiation therapy (nCRT), whereas patients having early cancer usually undergo total mesorectal excision, and for the selected patients, trans-anal excision [2]. Conventional MRI is also considered the first-line imaging modality for the evaluation of treatment response after nCRT to determine further treatment plan. Specifically, for selected patients achieving complete remission, deferred surgery with surveillance could be possible based on a watch and wait policy; and for patients with favorable therapeutic response, total mesorectal excision could be carried out due to clearance of mesorectal fascia [11]. In case of a bad prognostic factor like increased tumor volume on follow-up MRI after nCRT, prompt radical surgery could be considered without waiting for delayed radiation effect to the tumor. However, when distant metastases are found in follow-up abdomen and chest CT, curative surgery can be switched to palliative surgery combined with systemic chemotherapy.

Dynamic contrast-enhanced magnetic resonance imaging (DCE-MRI) is a quantitative imaging technique that measures perfusion parameters that reflect tumor angiogenesis [12]. Angiogenesis is a typical characteristic of tumor viability in numerous malignant tumors including rectal cancer [13,14,15]. DCE-MRI shows promising results in the visualization and quantitative assessment of tumor angiogenesis with perfusion parameters derived from pharmacokinetic modeling [2,16]. One such perfusion parameter is K^trans^, which refers to the rate of transfer of a contrast medium between blood and tissue [17]. In certain tumors, K^trans^ is thought to reflect vascular permeability, indicating its ability to determine disease progression and therapeutic response [18,19].

In rectal cancer, many studies have reported the efficacy of DCE-MRI for assessing the therapeutic response to nCRT in locally advanced rectal cancer [20,21,22,23,24]. To date, only a few studies have focused on the association between various prognostic factors and MR perfusion parameters in primary rectal cancer; however, the results have been inconsistent [2,16,17,25,26,27]. Thus, the aim of the present study was to investigate associations between MR perfusion parameters and various prognostic factors in patients with primary rectal cancer.

## 2. Materials and Methods

This retrospective study was approved by our institutional review board and informed consent from individual patients was waived.

### 2.1. Patients and Patient Selection Criteria

**Figure 1:**
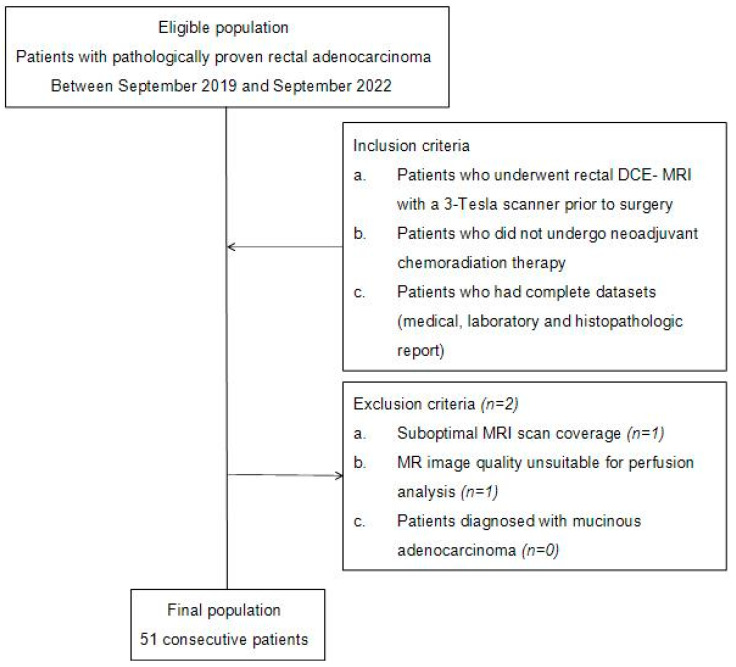
Flowchart of case accrual process.

Between September 2019 and September 2022, patients who had been proven to have rectal adenocarcinoma via colonoscopic biopsy and who satisfied our inclusion criteria were initially recruited for this study. The inclusion criteria were as follows: (1) patients who underwent rectal DCE-MRI with a 3-Tesla scanner before surgery; (2) patients who underwent subsequent surgical excision without nCRT; and (3) patients who had complete datasets including medical, laboratory, and histopathologic report. A total of 53 patients (33 men, 20 women) were eligible. Among them, 2 patients were excluded due to the following reasons: (1) a patient with suboptimal scan coverage (*n* = 1); (2) a patient whose MR image quality was not suitable for perfusion analysis (*n* = 1); and (3) a patient who had been proven to have mucinous adenocarcinoma via histopathologic study (*n* = 0). A total of 51 consecutive patients (31 men, 20 women; mean age, 69 years; range, 45–89 years) were thus included in the study. The case enrollment process is summarized in Figure 1.

### 2.2. MRI Technique

MRI examinations were carried out on a 3-Tesla MR scanner (Achieva TX; Philips, Best, Netherlands) using a torso coil (SENSE; USA Instruments, Gainesville, FL, USA). Antiperistaltic agents were not used. The scan protocols were as follows: T2-weighted imaging in 3 planes and axial T1-weighted 3-dimensional spoiled gradient echo (fast-field echo) imaging before and after contrast media injection. After the intravenous injection of 0.1 mmol/kg or 0.2 mL/kg of contrast agent (gadoterate meglumine, Clariscan, GE healthcare, Chicago, IL, USA) at a rate of 3.0 mL/s, 30 mL of normal saline was flushed. The temporal resolution of the DCE sequence was 3.6 s, and dynamic data acquisition was repeated 70 times for full coverage of the rectal cancer. The details of the scan parameters are given in Table 1.

### 2.3. Perfusion Parameter Measurement

The perfusion analyses were performed on a workstation (ISP, version 10.0, Philips, Best, Netherlands) equipped with a perfusion analysis tool (MR permeability). The MR permeability tool adopts an arterial input function (AIF) that is based on a population-based medium bi-exponential model. This function can be pre-selected according to the user selective injection duration as follows; short (less than 5 s), medium (between 5 and 10 s), and long (longer than 10 s) [28]. Two radiologists (with 18 years and 2 years of experience in interpreting rectal MRIs, respectively) read all the MR images for each patient. After determining the tumor border, the junior radiologist placed the region of interest (ROI) along the tumor border for each consecutive post-contrast T1WI image to cover the whole tumor volume [29]. The perfusion parameters were automatically calculated using the extended Toft model [28,30].

The perfusion parameters were as follows: forward volume transfer constant (K^trans^), fractional extracellular extravascular space (EES) volume (v_e_), plasma volume fraction (v_p_), and revere volume transfer constant (k_ep_). The K^trans^ was defined as the influx from the blood plasma to the EES. The k_ep_ was defined as the efflux from the EES to the blood plasma. The volume fractions of EES and blood plasma were represented as v_e_ and v_p_, respectively. The mean values were calculated as a representative by averaging each measured perfusion parameter values for the entire tumor [30].

### 2.4. Reference Standard for Prognostic Factors

Laboratory results, including CEA levels, were taken from patients’ medical records. Pathologic examinations were conducted by a dedicated pathologist who had 10 years of clinical experience in accordance with the TNM staging system [6]. Tumor differentiation grading was divided according to the 2019 WHO grading system based on the percentage of glandular formation throughout the tumor, with cut-offs of >95%, 50–95%, and <50% indicating well, moderately, and poorly differentiated, respectively [31].

Existence of EMVI, circumferential resection margin (CRM) status, and immuno-histochemical results including KRAS mutation, were also reviewed [6,7,10]. EMVI is the presence of tumor cells in the veins, beyond the proper muscle layer of the rectal wall. EMVI can provide a pathway for hematogenous dissemination of tumor cells, and therefore, is an independent risk factor for disease relapse and metastasis [32]. KRAS mutation, which is found in 30–50% of colorectal cancer patients, has been demonstrated to be a predictive biomarker of resistance to anti-epidermal growth factor receptor (EGFR) therapy. It is well known that KRAS mutation is associated with poor response to anti-EGFR therapy [10,33].

### 2.5. Statistical Analysis

The association between the perfusion parameters and the prognostic factors was assessed using a correlation test for continuous data between prognostic factors or t-tests for categorical data. In the case of a significant association, receiver operating characteristic (ROC) curve analyses were performed. An area under the ROC curve (AUC) was calculated and considered the diagnostic performance. The sensitivity, specificity, and accuracy were estimated under the optimal cut-off value. Correlation analysis was also performed to study the relationship between perfusion parameters and measurable prognostic factors. The MedCalc software for Windows (MedCalc Software version 20.113, Mariakerke, Belgium) was used for all the statistical analyses, and *p*-values less than 0.05 were considered significant.

## 3. Results

### 3.1. Patient Demographics

The mean duration between MRI and surgery was 9 days (range, 1–36 days). The average tumor size was 4.66 cm (range, 0.6–11 cm), and the average CEA level was 10.98 ng/mL (0.3–280 ng/mL). The location of the tumor was in the upper (*n* = 15), mid (*n* = 20), or lower rectum (*n* = 16). The tumor differentiation was composed of moderately differentiated (*n* = 45) and well-differentiated tumors (*n* = 6). A pathologic EMVI was observed in eight patients. KRAS mutations were observed in 22 patients. The detailed demographic data, including the tumor and node stage, of the study population are given in Table 2.

Patients with locally advanced rectal cancer (stage ≥ II) routinely underwent nCRT prior to surgical resection in our hospital. However, patients with colonic obstruction due to rectal cancer underwent surgery first without nCRT. Although it is controversial, 10 patients with good T3 (extramural growth < 5 mm) and node negative on MRI also underwent surgical resection first, then followed by adjuvant chemotherapy.

### 3.2. Comparison of Perfusion Parameters According to Prognostic Factors

**Figure 2:**
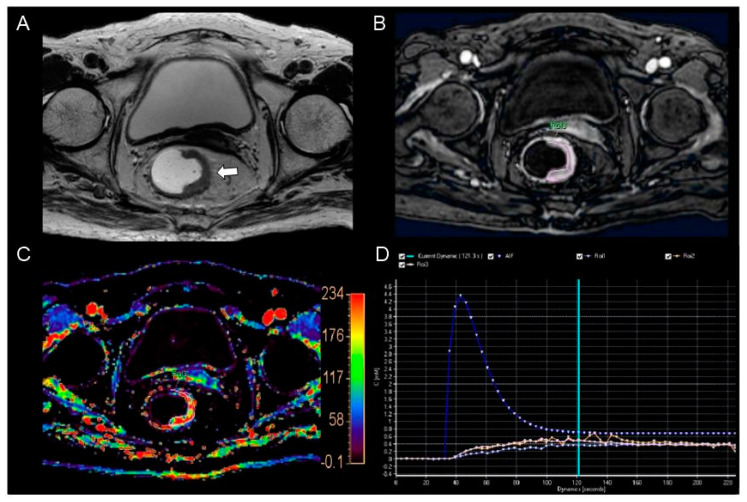
MR images from an 86−year−old woman with pathologically proven well−differentiated rectal adenocarcinoma. (**A**) The T2−weighted axial image shows an ulcero-infiltrative mass (arrow) in the left wall of the mid rectum. (**B**) The post−contrast axial 3−dimensional spoiled gradient echo image shows a relatively hyper−enhancement in the corresponding tumor. (**C**) The color−coded K^trans^ map shows a predominantly red color in the corresponding tumor. The calculated mean K^trans^ was 0.137 min^−1^. (**D**) The concentration time curve shows a plot of arterial input function (AIF, blue line), which was incorporated automatically with the population−based data and three plots from the consecutive three regions of interest (ROIs−1, 2, and 3) in the whole tumor (pale blue and orange lines).

**Figure 3:**
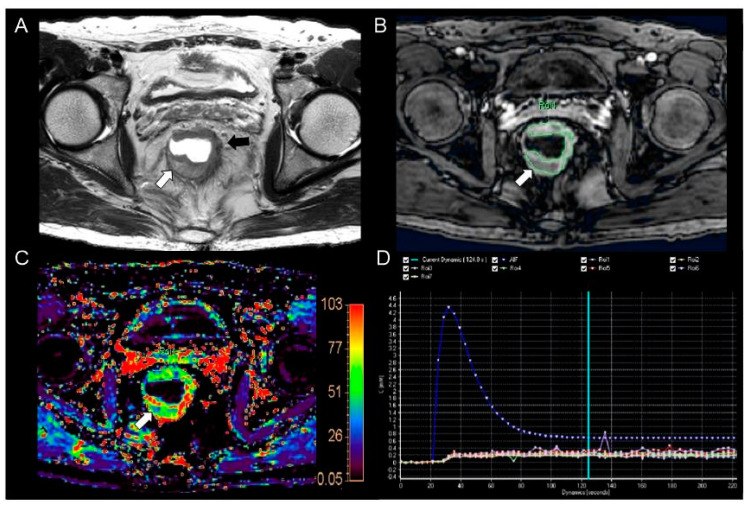
MR images from a 54−year−old man with pathologically proven moderately differentiated rectal adenocarcinoma. (**A**) The T2−weighted axial image shows eccentric wall thickening (black arrow) in the lower rectum. Focal high signal intensity is seen (white arrow) in the posterior portion of the tumor. The focal lesion was histologically revealed to be mucin accumulation in the tumor. (**B**) The post−contrast axial 3−dimensional spoiled gradient echo image shows a heterogeneous enhancement in the corresponding tumor. Relatively poor enhancement is seen in the mucin accumulation site (arrow). (**C**) The color-coded K^trans^ map shows a wide range of colors from purple to red in the corresponding tumor. Mixed blue and purple colors are seen in the mucin accumulated portion (arrow). The calculated mean K^trans^ was 0.063 min^−1^. (**D**) The concentration time curve shows a plot of arterial input function (AIF, blue line), which was implemented automatically with the population−based data and seven plots from the consecutive seven regions of interest (ROIs−1 to 7) in the whole tumor (different colored lines).

Among the perfusion parameters and prognostic factors, only K^trans^ and k_ep_ showed a significant difference by tumor differentiation—between the well-differentiated and moderately differentiated groups (K^trans^, 0.127 ± 0.032, 0.084 ± 0.036, *p* = 0.036; k_ep_, 0.623 ± 0.252, 0.415 ± 0.151, *p* = 0.005, respectively) (Figure 2 and Figure 3). Of the two perfusion parameters, only K^trans^ showed a significant AUC (0.838, 95% CI, 0.702–0.929, *p* < 0.0001). Under the cutoff value of 0.130, the estimated maximum accuracy, sensitivity, and specificity were 87% (41/47), 90% (38/42), and 60% (3/5), respectively. On the other hand, k_ep_ showed an AUC of 0.758 (95% CI, 0.616–0.868, *p* = 0.08), which was not statistically significant.

In addition, the TN stage did not show significant differences for four perfusion parameters (*p* > 0.05). The EMVI positive (*n* = 8) did not show a significant difference compared with the EMVI negative (*n* = 43) for four perfusion parameters (*p* > 0.05). The KRAS positive (*n* = 22) did not show a significant difference compared with the KRAS negative (*n* = 29) for four perfusion parameters (*p* > 0.05). The CRM positive status (*n* = 8) did not show a significant difference compared with the CRM negative status (*n* = 43) for four perfusion parameters (*p* > 0.05), either. The statistical results are given in Table 3.

In a subgroup analysis, the tumor size was also significantly different between the well-differentiated and moderately differentiated groups (2.82 ± 1.78 and 4.72 ± 2.26, respectively, *p* = 0.0496). However, the CEA levels were not significantly different from each other (1.86 ± 0.47 and 7.90 ± 7.67, respectively, *p* = 0.2243). The results are summarized in Table 4.

In terms of correlation coefficients, there was no significant correlation between the tumor size and K^trans^ (r = −0.081, 95% CI, −0.351–0.202, *p* = 0.585) or between the CEA level and K^trans^ (r = −0.143, 95% CI, −0.408−0.144, *p* = 0.327).

## 4. Discussion

We assessed the association between MR perfusion parameters and various prognostic factors for primary rectal cancer. Our results showed that K^trans^ only showed a significant difference according to tumor differentiation. Specifically, the value was higher in the well-differentiated group than in the moderately differentiated one. These results are contrary to those of previous studies [2,26]. Shen et al. reported that the K^trans^ value was lower in the well-differentiated group (*n* = 6) than in the moderately differentiated one (*n* = 21) (0.182 ± 0.153, 0.280 ± 0.067, respectively, *p* = 0.004) [2]. However, the values between the moderately and poorly differentiated groups (*n* = 13) were not significantly different (0.284 ± 0.068). Another study by Li et al. also showed that the K^trans^ value was higher in the poorly differentiated group (*n* = 41) than in the well- (*n* = 6) and moderately differentiated ones (*n* = 51) (0.92 ± 0.51, 0.53 ± 0.29, respectively, *p* < 0.001) [26].

The discrepancy between our observation and previous studies could be attributed to the difference in whole tumor coverage during the measurement process. A previous study only selected a representative image in each plane with no less than 1 cm^2^ to measure perfusion parameters [2]. Another previous study manually drew the ROI to cover the entire tumor while excluding internal necrosis and cystic portions [26].

From the viewpoint of the tumor environment, mucin production and internal necrosis are frequently seen in rectal cancer and constitute tumor heterogeneity [34,35]. Contrary to previous studies, the consecutive image slices bearing whole tumor portions were covered to measure the perfusion parameters of the entire tumor while including internal necrosis and cystic portions containing mucin in our study [26]. Based on our experience, the larger the tumor, the more heterogeneous the tumor environment. In fact, the tumor size was larger in the moderately differentiated group than in the well-differentiated one in our study (4.72 ± 2.26 cm, 2.82 ± 1.78 cm, respectively). Thus, the heterogeneous tumor environment would be more reflected in the present study than in previous ones in which only pure solid tumor portions were included.

We acknowledge that the tumor differentiation by itself affects the neo-vascularization of the tumor; that is, newly developed fragile and impaired vessels are more frequently seen in poorly differentiated tumors than in well-differentiated ones. As a result, higher permeability (a higher K^trans^ value) of the tumor is supposed to be observed in the more disorganized tumor environment with poorly differentiated tumor cells [2].

In terms of the therapeutic response to nCRT in locally advanced rectal cancer, K^trans^ showed a significant decrease in the responder (tumor-downstaged group), whereas it was unchanged in poor responders (tumor-non-downstaged group) (in responders, from 1.24 ± 0.53 to 0.76 ± 0.45, *p* = 0.0007; in non-responders, from 1.02 ± 0.53 to 0.87 ± 0.48, *p* = 0.2358) [6]. However, the mean K^trans^ values of the pre-CRT MRIs were not different from each other (*p* = 0.1393).

Although KRAS mutation status and EMVI did not show significant differences between the other perfusion parameters in our study, those factors showed a potential benefit in the literature [25,36,37,38]. KRAS mutation is an independent prognostic factor and is known to be resistant to chemotherapy in rectal cancer [36]. Thus, patients with the wild type (non-mutated KRAS) show a favorable response to chemotherapy compared to patients with the KRAS mutation. Our results correspond well with a previous study. Yeo et al. also observed that K^trans^ in a KRAS-mutated group showed no significant difference compared with a wild-type group (0.123 ± 0.032, 0.100 ± 0.039, respectively, *p* = 0.06) [17].

EMVI is also known to be a prognostic factor in rectal cancer. A previous study revealed that K^trans^ was higher in the EMVI-positive group than in the EMVI-negative group (1.08 ± 0.946, 0.542 ± 0.636, respectively, *p* = 0.02) [25]. In addition, K^trans^ combined with Ve showed a moderate accuracy (AUC, 0.715 in the validation cohort) for the pre-operative prediction of EMVI in rectal cancer. Another study also observed that a MR-detected EMVI (mrEMVI)-positive group showed a higher median K^trans^ value than a mrEMVI-negative group (0.74, 0.39, respectively, *p* < 0.01), and the AUC was 0.779 [37]. The discrepancy between those results and ours could be explained by the composition of the study population. In our study, most of the patients with T3 stage showed the EMVI negative except for three patients with bad T3 (extramural growth > 15 mm). Thus, no significant difference in K^trans^ could be attributed to the relatively small number of the EMVI positive (*n* = 8) compared to a large number of the EMVI positive in T3 or T4 stage in those studies (*n* = 28, *n* = 24) [25,37].

Regarding the future perspectives of quantitative imaging modalities including DCE-MRI, diffusion weighted imaging, texture analyses, and radiomics on rectal cancer, much evidence has been collected to support consensus statements beyond the expert opinions [38]. Particularly, the amount of research into MRI-based radiomics in locally advanced rectal cancer has increased. Several studies have shown that MRI radiomics are useful in predicting tumor response after nCRT [39,40,41]. In addition, radiomics can be combined with emerging artificial intelligence, so-called deep learning to create a new field of predicting treatment response and prognosis.

Several limitations should be acknowledged in this study. First, the small study population of the well-differentiated group should be considered. However, well-differentiated tumors are known to constitute 10% of rectal adenocarcinomas, and thus they were enrolled in this study at a reasonable rate. In contrast, poorly differentiated tumors, which are known to constitute 15% of adenocarcinomas, were absent in the present study. The reasons are pathological sampling bias associated with tumor heterogeneity and the inherent subjective nature of the WHO grading system that our pathologist followed [31]. Second, inter-observer agreement in the measurements of perfusion parameters were not evaluated because the data from the whole tumor volume were known to be most reproducible among various measurement methods including single ROI or several ROIs covering a small solid tumor portion in a quantitative analysis in rectal cancer [42]. Third, the range of reproducibility according to different vendors was known to be within 20%, which is an inherent limitation in DCE-MRI [43,44]. Last, external validation could not be conducted because acquisition of DCE-MRI is considered as a research tool and not adopted routinely, thus it is very limited in other hospitals even in the tertiary referral hospitals.

## 5. Conclusions

In conclusion, K^trans^ showed a significant difference according to tumor differentiation, which may be conducive to the prediction of prognosis in primary rectal cancer.

## Figures and Tables

**Table 1:** MR sequence parameters.

Parameters	T2-Weighted Axial, Sagittal, and Coronal	Pre-Contrast Axial 3D-Spoiled Gradient Echo	Post-Contrast Axial 3D-Spoiled Gradient Echo
TR (msec)	2500–3782	10.0	3.5
TE (msec)	90	1.6	1.2
Slice thickness (mm)	3, 5, 4	4	4
Slice gap (mm)	0.3, 1, 0.4	0	0
Matrix size	316 × 281, 356 × 331, 332 × 317	216 × 166	216 × 166
Flip angle (degree)	-	5, 15	8
FOV (mm × mm)	220 × 220	300 × 300	300 × 300
Acquisition time	2 min 15–25 s	10 s	4 min 12 s
Number of slices	26–30	18	18

TR, repetition time; TE, echo time; FOV, field of view.

**Table 2:** Demographic data of the study population.

Characteristic	Study Population (*n* = 51)
Tumor location	
Upper rectum	15 (29%)
Mid rectum	20 (39%)
Lower rectum	16 (31%)
Histological grade	
Well-differentiated	6 (12%)
Moderately differentiated	45 (88%)
Histologic tumor stage	
pT1	8 (16%)
pT2	13 (25%)
pT3	25 (49%)
pT4	5 (10%)
Pathologic lymph node stage	
N0	33 (65%)
N1	12 (23%)
N2	6 (12%)
EMVI	
Positive	8 (16%)
Negative	43 (84%)
CRM status	
Positive	8 (16%)
Negative	43 (84%)
KRAS mutation	
Positive	22 (43%)
Negative	29 (56%)

EMVI, extramural venous invasion; CRM, circumferential resection margin; KRAS, Kirsten-ras.

**Table 3:** Comparison of perfusion parameters according to prognostic factors.

Perfusion Parameters Prognostic Factors	K^trans^ (min^−1^, Mean ± SD)	*p* Value	k_ep_ (min^−1^, Mean ± SD)	*p* Value	v_e_ (Mean ± SD)	*p* Value	v_p_ (Mean ± SD)	*p* Value
T stage		0.7525		0.4767		0.3517		0.8902
pT1, pT2 (*n* = 21)	0.093 ± 0.046		0.457 ± 0.201		0.224 ± 0.148		0.018 ± 0.016	
pT3, pT4 (*n* = 30)	0.101 ± 0.067		0.421 ± 0.160		0.264 ± 0.152		0.018 ± 0.015	
N stage		0.5373		0.4179		0.3010		0.4043
N0 (*n* = 33)	0.102 ± 0.069		0.450 ± 0.194		0.231 ± 0.147		0.019 ± 0.018	
N1-2 (*n* = 18)	0.092 ± 0.038		0.408 ± 0.141		0.278 ± 0.155		0.016 ± 0.009	
EMVI		0.7254		0.5961		0.4213		0.5178
Positive (*n* = 8)	0.094 ± 0.028		0.405 ± 0.146		0.291 ± 0.160		0.016 ± 0.007	
Negative (*n* = 43)	0.099 ± 0.064		0.441 ± 0.183		0.239 ± 0.149		0.018 ± 0.017	
KRAS mutation		0.2793		0.6904		0.6747		0.6253
Positive (*n* = 22)	0.087 ± 0.040		0.418 ± 0.170		0.237 ± 0.177		0.016 ± 0.016	
Negative (*n* = 29)	0.105 ± 0.071		0.438 ± 0.178		0.256 ± 0.132		0.019 ± 0.016	
Tumor differentiation		0.0360		0.0053		0.3333		0.7004
Well (*n* = 6)	0.127 ± 0.032		0.623 ± 0.252		0.327 ± 0.206		0.021 ± 0.018	
Moderately (*n* = 45)	0.084 ± 0.036		0.415 ± 0.151		0.235 ± 0.142		0.018 ± 0.016	
CRM status		0.9337		0.3737		0.4742		0.8316
Positive (*n* = 8)	0.097 ± 0.045		0.384 ± 0.099		0.287 ± 0.168		0.017 ± 0.010	
Negative (*n* = 43)	0.099 ± 0.062		0.445 ± 0.187		0.240 ± 0.148		0.018 ± 0.016	

EMVI, extramural venous invasion; KRAS, Kirsten-ras; CRM, circumferential resection margin.

**Table 4:** Comparison of prognostic factors according to tumor differentiation.

Prognostic Factors	Well-Differentiated Group (*n* = 6) (Mean ± SD)	Moderately Differentiated Group (*n* = 45) (Mean ± SD)	*p* Value
CEA level (ng/mL)	1.86 ± 0.47	7.90 ± 7.67	0.2243
Tumor size (cm)	2.82 ± 1.78	4.72 ± 2.26	0.0496

CEA, carcinoembryonic antigen.

## Data Availability

The data presented in this study are available on request from the corresponding author. The data are not publicly available due to patients’ privacy.
